# Selfing in epiphytic bromeliads compensates for the limited pollination services provided by nectarivorous bats in a neotropical montane forest

**DOI:** 10.1093/aobpla/plae011

**Published:** 2024-02-26

**Authors:** Stephanie Núñez-Hidalgo, Alfredo Cascante-Marín

**Affiliations:** Sistema de Estudios de Posgrado, Universidad de Costa Rica, San Pedro de Montes de Oca, 11501-2060 San José, Costa Rica; Escuela de Biología y Centro de Investigación en Biodiversidad y Ecología Terrestre (CIBET), Universidad de Costa Rica, San Pedro de Montes de Oca, 11501-2060 San José, Costa Rica

**Keywords:** Breeding systems, Bromeliaceae, chiropterophily, Costa Rica, pollinator limitation, reproductive assurance

## Abstract

**Abstract**. Plants with specialized pollination systems frequently exhibit adaptations for self-pollination, and this contradictory situation has been explained in terms of the reproductive assurance function of selfing. In the neotropics, several plant lineages rely on specialized vertebrate pollinators for sexual reproduction, including the highly diverse Bromeliaceae family, which also displays a propensity for selfing. Thus far, the scarce evidence on the role of selfing in bromeliads and in other neotropical plant groups is inconclusive. To provide insights into the evolution and persistence of self-fertilization in the breeding systems of Bromeliaceae, we studied four sympatric epiphytic species from the genus *Werauhia* (Tillandsioideae) in Costa Rica. We documented their floral biology, pollination ecology and breeding systems. We estimated the contribution of selfing by comparing the reproductive success between emasculated flowers requiring pollinator visits and un-manipulated flowers capable of selfing and exposed to open pollination across two flowering seasons. The studied species displayed specialized pollination by nectar-feeding bats as well as a high selfing ability (auto-fertility index values > 0.53), which was attained by a delayed selfing mechanism. Fruit set from natural cross-pollination was low (<26% in both years) and suggested limited pollinator visitation. In line with this, we found a very low bat visitation to flowers using video-camera recording, from 0 to 0.24 visits per plant per night. On the contrary, the contribution of selfing was comparatively significant since 54–80% of the fruit set from un-manipulated flowers can be attributed to autonomous self-pollination. We concluded that inadequate cross-pollination services diminished the reproductive success of the studied *Werauhia*, which was compensated for by a delayed selfing mechanism. The low negative effects of inbreeding on seed set and germination likely reinforce the persistence of selfing in this bromeliad group. These results suggest that selfing in bat-pollinated bromeliads may have evolved as a response to pollinator limitation.

## Teaser

Many flowering plants have the ability to produce viable seeds through autonomous self-pollination, thus obviating the need for animal pollinators. This is regarded as beneficial when conditions for cross-pollination are not optimal, however, there is insufficient evidence to support this notion for neotropical plants. In a group of four bromeliads from the genus Werauhia (Pineapple family) pollinated by nectarivorous bats, we found a high ability to self-pollinate autonomously. This occurred at the end of the flower´s life and accounted for most (54–80%) of the sired fruits. Contrary to that, cross-pollination by bats was low and resulted in a reduced number of fruits (<26%). The limited pollination services provided by bats may be the result of their diminished diversity and abundance in the studied montane forest. These findings support the idea that in conditions of pollinator scarcity or unreliability, plants can resort to self-pollination as a mechanism of reproductive assurance.

## Introduction

Selfing or the ability to self-fertilize in plants, is a relatively common reproductive strategy among angiosperms (~20%) (Barrett, 2002), and in several species, the floral mechanisms that facilitate selfing also co-exist with specialized pollination systems in a mixed mating system (Fenster and Martén-Rodríguez, 2007). The maintenance of selfing as part of mixed mating systems in plants is largely attributed to its benefits as a ‘reproductive assurance’ mechanism in the face of unreliable cross-pollination (Mallick, 2001; Herlihy and Eckert, 2002; Kalisz *et al.*, 2004; Moeller and Geber, 2005; Moeller, 2006; Fenster and Martén-Rodríguez, 2007; Zhi-Quiang and Quing-Jun, 2008; Martén-Rodríguez and Fenster, 2010; Busch and Delph, 2012; Jones *et al.*, 2013).

The ability to self-fertilize requires the loss of self-incompatibility mechanisms and the existence of floral biology adaptations to facilitate the autonomous deposition of self-pollen onto the stigma. These mechanisms include the absence of intra-floral herkogamy (Webb and Lloyd, 1986) and dichogamy (Bertin and Newman, 1993). The establishment of self-fertilization is also contingent on the absence or reduced inbreeding depression effects (Charlesworth and Charlesworth, 1987; Eckert *et al.*, 2006). In addition, for selfing to provide reproductive assurance, the reproductive success of a species must be constrained by pollen availability or pollinator services (i.e. pollen limitation) (Eckert *et al.*, 2006), and it should not incur in pollen and ovules discount (Knight *et al.*, 2005).

The Bromeliaceae family is a very diverse group of monocotyledonous plants, almost entirely restricted to the American continent (Benzing, 2000). They contribute significantly to the floristic diversity of vascular epiphytic floras in the Neotropics (Cascante-Marín and Nivia-Ruíz, 2013). Bromeliads possess specialized pollination systems that involve vertebrate pollinators (hummingbirds and nectarivorous bats) and insects to a lesser degree, mainly bees (Benzing, 2000; Kessler and Krömer, 2000; Aguilar-Rodríguez *et al.*, 2019a; Kessler *et al.*, 2020). Even though most bromeliads exhibit adaptations for cross-pollination, nearly two-thirds of the species investigated for their reproductive systems are capable of selfing. This is more frequent in the subfamilies Tillandsioideae and Pitcairnioideae (Cascante-Marín and Núñez-Hidalgo, 2023). However, little attention has been paid to detailed studies of selfing mechanisms and their adaptive value in neotropical plants as a whole. Previous works (Wendt *et al.*, 2002; Matallana *et al.*, 2010) have proposed that the prevalence of selfing among bromeliads represents a reproductive isolation strategy (*sensu*Levin, 1971) to minimize the negative effects of hybridization in sympatry. Nevertheless, the evidence supporting either hypothesis (‘reproductive assurance’ or ‘reproductive isolation’) is inconclusive in this important group of monocots (Cascante-Marín and Núñez-Hidalgo, 2023).

The mechanisms of selfing vary with regard to the precise moment of its occurrence, and they define the role of selfing, which has evolutionary consequences for plant fitness (Lloyd, 1992; Lloyd and Schoen, 1992; Brys and Jacquemyn, 2011). Selfing may occur either before anthesis (prior selfing), during anthesis when the flower is exposed to cross-pollination (competing selfing), or at the end of its life (delayed selfing) (Schoen and Lloyd, 1992). Selfing that occurs late in the flower’s life (‘delayed selfing’), when the possibility of cross-pollination has passed, is likely to result in reproductive assurance (Fenster and Martén-Rodríguez, 2007; Goodwillie and Weber, 2018). Delayed selfing does not interfere with pollen pick-up by pollinators or with the stigma’s receipt of crossed pollen, hence decreasing pollen and ovule discounting, respectively (Lloyd, 1992; Herlihy and Eckert, 2002).

This study seeks to further our understanding of the sexual reproductive systems of neotropical plants, particularly the evolution and maintenance of selfing in the Bromeliaceae family. Using information from floral biology, pollination ecology, and breeding systems of species from the genus *Werauhia* J. R. Grant in the subfamily Tillandsioideae, we intend to provide insights into the ecological causes for the persistence and predominance of self-fertilization in this plant group. *Werauhia* is proposed as a monophyletic group (Barfuss *et al.*, 2005) and is represented by one hundred recognized species (Gouda and Butcher, 2016 and cont. updated) of epiphytic life-form and distributed mainly on the mountains of southern Central America (Costa Rica and Panama) (Grant, 1995; Morales, 2003). Previous studies in *Werauhia* indicate the presence of specialized pollination systems involving nocturnal nectarivorous bats (Aguilar-Rodríguez *et al.*, 2019a) and hummingbirds (Lasso and Ackerman, 2004), as well as high selfing ability in *W. gladioliflora* (Cascante-Marín *et al.*, 2005; Tschapka and von Helversen, 2007), *W. nutans* and *W. noctiflorens* (Aguilar-Rodríguez *et al.*, 2019b), and *W. sintenisii* (Lasso and Ackerman, 2004).

We studied four *Werauhia* species that coexist simpatrically in a Costa Rican montane forest and characterized their floral biology (herkogamy, dichogamy, anthesis and senescence behaviour of flowers), pollination system (identified the main pollinators and their visitation rates) and the components of their reproductive systems (i.e. self-compatibility, selfing capacity and presence of agamospermy). We also evaluated the presence of inbreeding depression in self-fertilized progeny and estimated the contribution of selfing to reproductive success in natural conditions during two flowering episodes. We predict that our study species will exhibit high self-compatibility and selfing capacity, and if selfing acts as a safeguard against unpredictable cross-pollination (i.e. reproductive assurance), then it should occur at the end of the flower life (‘delayed selfing’) (sensu Goodwillie and Weber, 2018).

## Materials and Methods

### Study site

This study was conducted at Cerros de (Hills of) La Carpintera Protective Zone in Costa Rica, between 2018 and 2021. The area comprises a small mountain formation in the eastern region of the Central Valley of the country (9º52’–9º54” N; 83º57’–84º00’ W; 1500–1850 m asl). The site comprises 2396 hectares covered by patches of primary forest interspersed with late secondary forest, and pastures (Sánchez *et al.*, 2008). The rainfall regime is seasonal, with a well-defined dry season from December to April. The site has a rich epiphytic flora and bromeliads are represented by 28 species from the genera *Aechmea* (1 spp.), *Catopsis* (3), *Guzmania* (3), *Pitcairnia* (1), *Racinaea* (2), *Tillandsia* (11), *Vriesea* (1), and *Werauhia* (6) (Sánchez *et al.*, 2008).

### Study species

**Figure 1.**
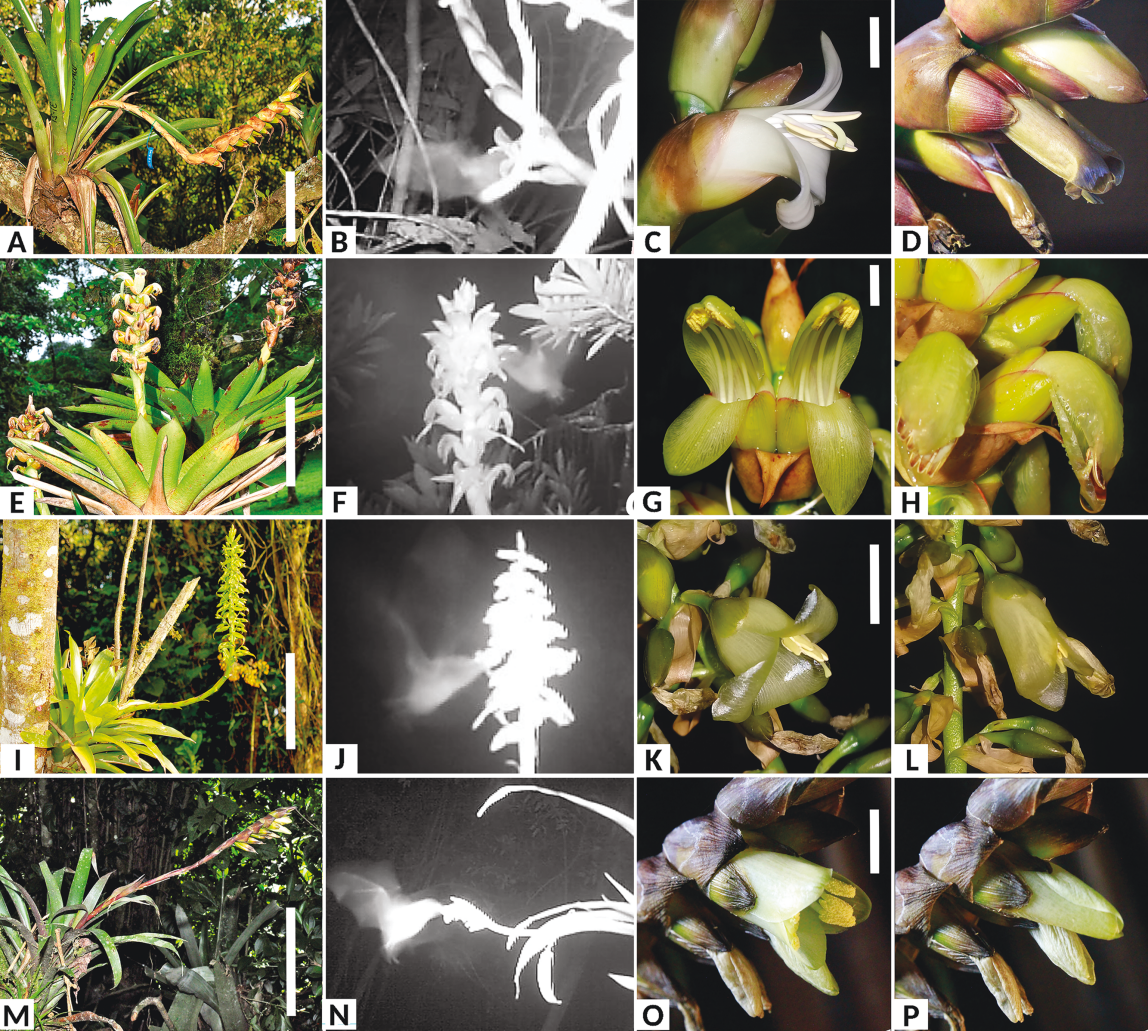
Studied species of *Werauhia* (Bromeliaceae: Tillandsioideae) in a montane forest, Cerros La Carpintera, Costa Rica. (A–D) *W. ampla*, (E–H) *W. nephrolepis*, (I–L) *W. pedicellata*, (M–P) *W. subsecunda*. (B, F, J, N) Night-vision images of bats visiting inflorescences of the studied species and recorded with video camera traps. (C, G, K, O) Flowers in anthesis. (D, H. L, P) Senescent flowers whose corollas have lost turgor. Scale bars = 10 cm (A, E, I, M) and 1.0 cm (C, G, K, O).

We selected the more abundant *Werauhia* species at the study site: *W. ampla*, *W. nephrolepis, W. pedicellata*, and *W. subsecunda* (Fig. 1). These are small to medium size and tank-forming bromeliads that develop a single spiked or compound inflorescence per rosette. *Werauhia* species are distinguished by having flowers with nocturnal anthesis, zygomorphic corollas with dull coloration (white or greenish), basal appendages of petals with the dactyloid divided apex, and a cupular-shaped stigma without papillae (Grant, 1995). The joint flowering period of the four species extends from November to August and shows significant inter-specific temporal displacement (Cascante-Marín *et al.*, 2017). Voucher specimens are deposited in the Luis Fournier O. Herbarium (USJ) at the University of Costa Rica (*W. ampla* USJ-100246, *W. nephrolepis* USJ-105232, *W. pedicellata* USJ-106525, and *W. subsecunda* USJ-111865).

### Floral biology

We documented nine floral traits for each species: (i) number of flowers per inflorescence, (ii) floral display (number of flowers open per day), (iii) colour of the inflorescence bracts (peduncle, primary and floral bracts), (iv) corolla colour and shape (campanulate or bilabiate), (v) stigma and anthers position relative to the corolla mouth, (vi) stigma-anthers separation or herkogamy, (vii) anthesis time and flower longevity, (viii) time of anther dehiscence and stigma receptivity or dichogamy, and (ix) mechanism of flower senescence. We tested stigma receptivity with a peroxidase test (King, 1960; Kearns and Inouye, 1993), using the presence of bubbling (observed with a 20× hand magnifying glass) on the stigmatic surface as an indicator of enzymatic activity.

We recorded the emission of floral volatile compounds through an organoleptic test (i.e. smelling the open flower and noticing any fragrance). Floral nectar volume and sugar concentration were measured in flowers from plants kept in a shade house at the study site. Before anthesis, flowers were isolated to prevent nectar consumption by floral visitors. Using glass capillary tubes, the accumulated volume was measured 2–4 h after anthesis. A handheld refractometer (Bellingham and Standley Ltd., UK) was used to estimate the sugar concentration in Brix degrees.

### Floral visitors and visitation frequency

We recorded the flower visitors to each bromeliad species in the forest with six video camera traps (Trophy cam, model 119476, Bushnell Corporation, Kansas, USA), during the flowering peaks of 2019, 2020, and 2021. The cameras were set to record 15-second-long videos when activated, followed by a period of 30 s of inactivity, during the day and night. At each focal plant, the complete flowering period of an inflorescence was monitored. Only in a few cases, it was interrupted due to battery depletion. The video analysis included: (i) number of visits, (ii) visitor identity (e.g. bats, hummingbirds, others), (iii) time and duration of each visit, and (iv) visitor behaviour (i.e. whether it contacted the anthers or stigma). The visitation rate per night for the most frequent visitors was determined by dividing the total number of recorded visits by the number of nights monitored each year.

To corroborate the chiropterophyllous transport of pollen, we captured bats to examine if they were carrying pollen from the studied species. We placed six mist nets (9 × 2.5 and 3 × 2.5 m) once or twice a week between January and February 2020, from 16:00 to 22:00 h, in sites considered as ‘passage zones’ for bats (Wilson *et al.*, 1996) and near flowering individuals of the studied species. This sampling only included the flowering period of *W. ampla* and *W. subsecunda*. The captured bats were identified following the taxonomic keys of York *et al.* (2019). Pollen was obtained from the top of the head and snout (cheeks-nose) using transparent adhesive tape. The piece of tape with pollen was attached to a microscope glass slide and a sampling area of 4.6 cm^2^ was visually scanned under a light microscope in the laboratory. We used a reference pollen collection from the study site to identify the pollen grains carried by the bats.

### Controlled pollination treatments and breeding systems evaluation

We conducted controlled pollinations on 73 plants (17 *W. ampla*, 15 *W. nephrolepis*, 16 *W. pedicellata,* and 25 *W. subsecunda*) kept in a shade house at the study site, from September 2018 to July 2019. We performed four pollination treatments: (i) manual self-pollination, (ii) manual cross-pollination, (iii) pollinator exclusion (autonomous selfing), and (iv) emasculation (test of agamospermy). The agamospermy test included stigma removal to avoid unnoticed contamination, this treatment did not affect further floral anthesis. Hand pollinations were conducted 1‒2 h after anthesis and flowers from all treatments were bagged until their senescence. All treatments were performed on each plant and randomly assigned to flowers in the same inflorescence. Fruit development was monitored in a monthly basis and the proportion of developed fruit in each treatment was calculated before fruit dehiscence.

The components of the reproductive systems were estimated using the parameters described by (Cascante-Marín and Núñez Hidalgo, 2023): (1) the *self-compatibility index*: SCI = Pa/ Px (Lloyd and Schoen, 1992), the *auto-fertility index*: AFI = Ps/ Px (Lloyd and Schoen, 1992), and the *agamospermy index*: AGI = Pag/ Px (Riveros *et al.*, 1996). For all indices: Pa = proportion of fruits after hand self-pollination, Pag = proportion of fruits after flower emasculation, Ps = proportion of fruits from flowers excluded from visitors and Px = proportion of fruits after hand cross-pollination.

### Reproductive success and inbreeding depression

We estimated the reproductive success per pollination treatment as the mean number of seeds per fruit in a sample of 8–52 fruits per treatment and species. Potential effects of inbreeding depression at the population level were tested by comparing seed production and seed germination capacity between manually self- and cross-pollination treatments. We conducted a germination test using seeds from 8 to 46 fruits per treatment (8–12 plants per species). Seeds were mixed and a sample of 480 seeds per treatment was distributed among 12 replicates of 40 seeds placed on wet towel paper in glass Petri dishes under lab conditions. As control, a similar number of seeds from open pollinated fruits were germinated. To avoid fungal contamination, we applied a commercial fungicide (Vitabax 40 WP) at the beginning of the experiment. The seeds were monitored and wetted (if necessary) twice a week and the number of germinated seeds recorded for two months. We considered a seed germinated when the radicle emergence from the seed coat was noticeable.

We performed an ANOVA test to detect significant differences in mean seed production between treatments per species and, after a significant result, we conducted post hoc pairwise comparisons (Tukey’s HSD test). Differences in mean cumulative percent of germinated seeds among treatments (self- and cross-pollinated, and natural pollination) for each species were evaluated using a non-parametric Kruskall–Wallis test (Zar, 2010). We used the Wilcoxon test for paired comparisons between treatments when significant differences were detected and applied a Bonferroni’s correction (Zar, 2010). Analyses were carried out using the built-in statistical functions available in the R software platform (R Core Team, 2023).

The reduction in fitness of selfed progeny was estimated with the *Inbreeding depression index* (Charlesworth and Charlesworth, 1987): IDI = 1 – (Ws/ Wo), where Ws = mean number of seeds per fruit or percentage of germinated seeds from manual selfing and Wo = mean number of seeds per fruit or proportion of germinated seeds from manual outcrossing. An ID*I*-value = 0 indicates the absence of inbreeding depression, while an ID*I* value = 1 indicates strong inbreeding depression.

### Reproductive assurance

To estimate the contribution of selfing to reproductive success, we compared the fruit set between emasculated and intact flowers under open pollination conditions in two consecutive flowering seasons. We emasculated 474 flowers from 13 to 42 plants per species in 2020 and 975 flowers from 31 to 53 plants per species in 2021. As control group, a similar number of intact flowers were selected in the same plants. Since plants from the studied species usually do not reproduce in consecutive years, the groups of manipulated plants differed in both years. Using an aluminium ladder, we included plants on host-trees within reach of six meters in height.

Emasculation was conducted in the afternoon (14–17 h) before floral anthesis, swollen flower buds in pre-anthesis were carefully open with a pair of tweezers and the anthers removed. This manipulation did not alter the floral anthesis. In the case of species with a high floral display per night, usually > 1 flower (*W. nephrolepis* and *W. pedicellata*), all flowers in anthesis were emasculated to avoid the possibility of geitonogamy. Fruits from emasculated flowers indicates a successful pollinator visit, whereas fruits from intact flowers may include both autonomous self- and cross-pollination.

We estimated the probability of fruit set between treatments (emasculated vs. control) with a generalized linear model (GLM) using a binomial distribution (link = ‘logit’) and a dichotomous response variable (success vs. failure). The model included as predictor variables: ‘treatment’, ‘year’, and their interaction, with categories ‘emasculated’ and ‘year 2020’ as reference. The model was estimated with the base package of the platform R (R Core Team, 2023). The Hosmer and Lemeshow test (*ResourceSelection* package; Lele *et al.*, 2019) evaluated the fit of the logistic model to the data. For those significant variables, we estimated the ‘odds ratio’ between the reference and respective categories of each variable and its 95% confidence limits.

The contribution of selfing to the reproductive success (i.e. fruit set) of each species per year was calculated using the *Reproductive Assurance Index* (Schoen and Lloyd, 1992): RAI = (Pi ‐ Pe)/ Pi; where Pi is the proportion of fruits from intact flowers and Pe is the proportion of fruits from emasculated flowers. Selfing contributes to reproduction if the RAI-value is greater than zero; when multiplied by 100, it indicates its relative contribution to the total fruit set. We also estimated the RAI using data on seed set from the 2021 season. For this, we counted the number of seeds in a sample of 16–32 fruits per treatment from each of the four studied species.

## Results

### Floral biology

Mean flower production per inflorescence varied from eight flowers in *W. subsecunda* to 55 flowers in *W. pedicellata*. Depending on the species, one to several flowers open per night, with *W. ampla* and *W. subsecunda* being less susceptible to geitonogamy, both species mostly open one flower per night (Table 1). All species released floral volatiles reminiscent of fermented fruits or garlic scents, whereas nectar production varied in terms of volume (11.9–598.1 μL) and concentration (8–18°Brix) per flower (Table 1).

**Table 1.** Floral biology traits of four sympatric *Werauhia* species (Bromeliaceae: Tillandsioideae) from a montane forest, Cerros La Carpintera, Costa Rica.

Floral trait	*W. ampla*	*W. nephrolepis*	*W. pedicellata*	*W. subsecunda*
Flowers per inflorescence—mean ± SD (range, sample size)	13.3 ± 3.6 (7–24, 45)	26.7 ± 6.1 (9–42, 42)	54.7 ± 29.6 (20–145, 42)	8 ± 1.8 (3–13, 50)
Floral display (open flowers per da)—mean ± SD (range, sample size)	1 (rarely 2) (37)	6.5 ± 2.5 (2–14, 37)	5.4 ± 2.7 (2–12, 32)	1 (rarely 2 or 3) (62)
Colour of peduncle, primary, and floral bracts at anthesis	Green to brown	Greenish	Green with reddish stripes	Green
Anthesis time	Late afternoon 15–17:30 h (*n* = 72)	Late afternoon 16–17:30 h (*n* = 71)	Late afternoon 16‒18:00 h (*n* = 91)	Late afternoon 15:30–17:00 h (*n* = 56)
Flower longevity	24 h (*n* = 58)	6–8 h (*n* = 55)	16–19 h (*n* = 31)	15–17 h (*n* = 32)
Corolla shape	Campanulate	Bilabiate	Campanulate	Campanulate
Corolla colour	White-green and suffused with purple toward the petals apex	White-greenish	White-translucent	White-greenish
Herkogamy type, (anters-stigma separation, sampled flowers)	Absent or approach type, stigma curved (2–5 mm, *n* = 58)	Absent, stigma curved (*n* = 55)	Absent or approach type (1.5‒2 mm, *n* = 31)	Mostly absent (*n* = 32)
Dichogamy type (temporal separation, sampled flowers)	Protogyny, incomplete (5‒135 min, 58)	Protogyny, incomplete (10‒60 min, 55)	Protogyny, incomplete (20‒75 min, 31)	Protogyny, incomplete (5‒70 min, 32)
Emission of floral scents (organoleptic test)	Slightly perceptible, fermented fruits	Perceptible, garlic and fermented fruits	Perceptible, garlic	Perceptible, fermented fruits
Nectar volume (μl) per flower—mean ± SD, [range], (sample size)	598.1 ± 217.2 [184.2–952.9] (33 fl/4 ind)	327.7 ± 199 [30–574.8] (30 fl/6 ind)	11.9 ± 10.0 [1–50.5] (33 fl/11 ind)	35.3 ± 29 [5–82.5] (20 fl/5 ind)
Nectar concentration (ºBrix)—mode, range (sample size)	17, 12–18 (33 fl./4 ind.)	12, 8–14 (30 fl./6 ind.)	12, 3–13 (33 fl./11 ind.)	12, 8–13 (20 fl./5 ind.)

In all studied species, reproductive organs were exposed to pollinators, the stigma and anthers projecting from or close to the corolla mouth (Fig. 1). Herkogamy was absent in *W. nephrolepis* and *W. subsecunda,* but variable in *W. ampla* and *W. pedicellata,* with some plants developing flowers with approach herkogamy (i.e. the stigma longer than the anthers) (Fig. 1C and 1K). Flowers of *W. ampla* and *W. nephrolepis* were distinguished by the upper portion of the style curving downward and away from the anthers (Fig. 1C). The four species showed incomplete protogyny. The stigma receptivity occurred early, sometimes even at the bud stage preceding anthesis, but soon it overlapped with pollen presentation. Temporal separation between female and male function varied within and between species by up to 2 h (Table 1).

Flowers exhibited late-afternoon anthesis (15:00–18:00 h), remaining fully open at night and for a period from 8 h in *W. nephrolepis* up to 24 h in *W. ampla* (Table 1). Flower senescence followed a similar pattern among the studied species, at the end of the flowerʼs life, the corolla loses its turgor and collapses (Fig. 1 and see Supporting Information—Videos S1 to S3). In the absence of herkogamy, the constriction of the petals brings the anthers with remaining pollen grains into contact with the stigma, which is still receptive and has accumulated a viscous fluid in the cupular stigmatic lobes. In *W. ampla* and *W. nephrolepis*, nectar dripping on the lower petal may remove pollen and deposit it on the stigma, increasing the likelihood of autonomous self-pollination.

### Pollinators and floral visitors

The video recording data comprised 454 nights and 1448 monitored flowers (see Supporting Information**—**Table S1). Bats visited the studied *Werauhia* on 33 occasions, usually between 19:00–23:00 h and 01:30–03:30 h, and each visit to a flower lasted around 2 s. The video images did not allow a precise identification of the bat species, but they revealed contact between the batʼs head and the flowerʼs reproductive organs (Fig. 1 and see Supporting Information—Videos S4–S7). Overall, the visitation rate per night per plant was quite low (0.07 visits) and varied among years and species from 0 to 0.24 (see Supporting Information—Table S1). In a few events, the video cameras were activated at night, but no activity was documented, which suggests the possibility of unrecorded visits.

Sporadic visits by the hummingbird *Lampornis calolaemus* (Trochilidae) to flowers of *W. ampla, W. nephrolepis,* and *W. pedicellata* were also video*-*recorded during the late afternoon at the beginning of flower anthesis (16:50–17:20 h) and the following morning (6:00–8:00 h) when flowers were wilting. A nocturnal and arboreal mouse from genus *Reithrodontomys* (Rodentia: Cricetidae) was occasionally recorded visiting flowers of *W. ampla* and *W. nephrolepis*. Stingless bees (*Trigona* sp., Apidae) were seen on flowers of *W. ampla* and *W. nephrolepis* collecting pollen from the anthers in the following day of anthesis.

During the mist-netting sampling of eight nights and with an effort of 675 m^2^/h, we captured 46 bats from nine genera. Pollen from the studied *Werauhia* species was recovered from three (out of five) captured individuals of the nectarivorous leaf-nosed bats *Hylonycteris underwoodi* and from the single capture of *Glossophaga soricina* (see Supporting Information**—**Table S2). Pollen counts varied between 7 and 5250 grains per sampled individual. Additional pollen recovered from the bats mainly belonged to the shrubby epiphytic nightshades: *Merinthopodium neuranthum* and *Schultesianthus leucanthus* (Solanaceae) (see Supporting Information**—**Table S2).

### Breeding systems

Hand self- and cross-pollinations resulted in high percentages (>75%) of fully developed fruits, except in *W. pedicellata* (50% and 58.1%, respectively) (Table 2). Fruit set from autonomous selfing was higher for *W.subsecunda* (76.7%) and *W. nephrolepis* (71.1%) and moderate in *W. ampla* (43.3%) and *W. pedicellata* (31.1%). The breeding systems of the four *Werauhia* species are characterized by high values of self-compatibility (SCI = 0.86–1.14), with relatively high values of self-fertility (AFI = 0.53–1.00), which indicate a high ability to self-pollinate by autonomous means. The agamospermy index suggested a very low degree of potential apomixis in *W. nephrolepis* and *W. pedicellata* (AGI = 0.11 and 0.06, respectively) (Table 2).

**Table 2.** Results of controlled pollination treatments and values of indexes that describe the breeding systems of four epiphytic *Werauhia* species (Bromeliaceae: Tillandsioideae) in a montane forest, Cerros La Carpintera, Costa Rica. Data are fruit percentages (%) and in parenthesis the number of developed fruits/manipulated flowers.

Experimenal variable	*W. ampla*	*W. nephrolepis*	*W. pedicellata*	*W. subsecunda*
Number of plants (N)	17	15	16	25
Manual self-pollination	75.0% (12/16)	100% (36/36)	50% (15/30)	87.5% (14/16)
Manual cross-pollination	82.4 % (14/17)	94.3 % (33/35)	58.1 % (18/31)	76.5 % (13/17)
Autonomous self-pollination	43.3% (13/30)	71.1% (32/45)	31.1% (14/45)	76.7% (23/30)
Agamospermy	0 (0/19)	10.5% (4/38)	3.4% (1/29)	0 (0/17)
Self-compatibility index (SCI)	0.91	1.06	0.86	1.14
Auto-fertility index (AFI)	0.53	0.75	0.54	1.00
Agamospermy index (AGI)	0.00	0.11	0.06	0.00

### Reproductive success and inbreeding depression

**Figure 2.**
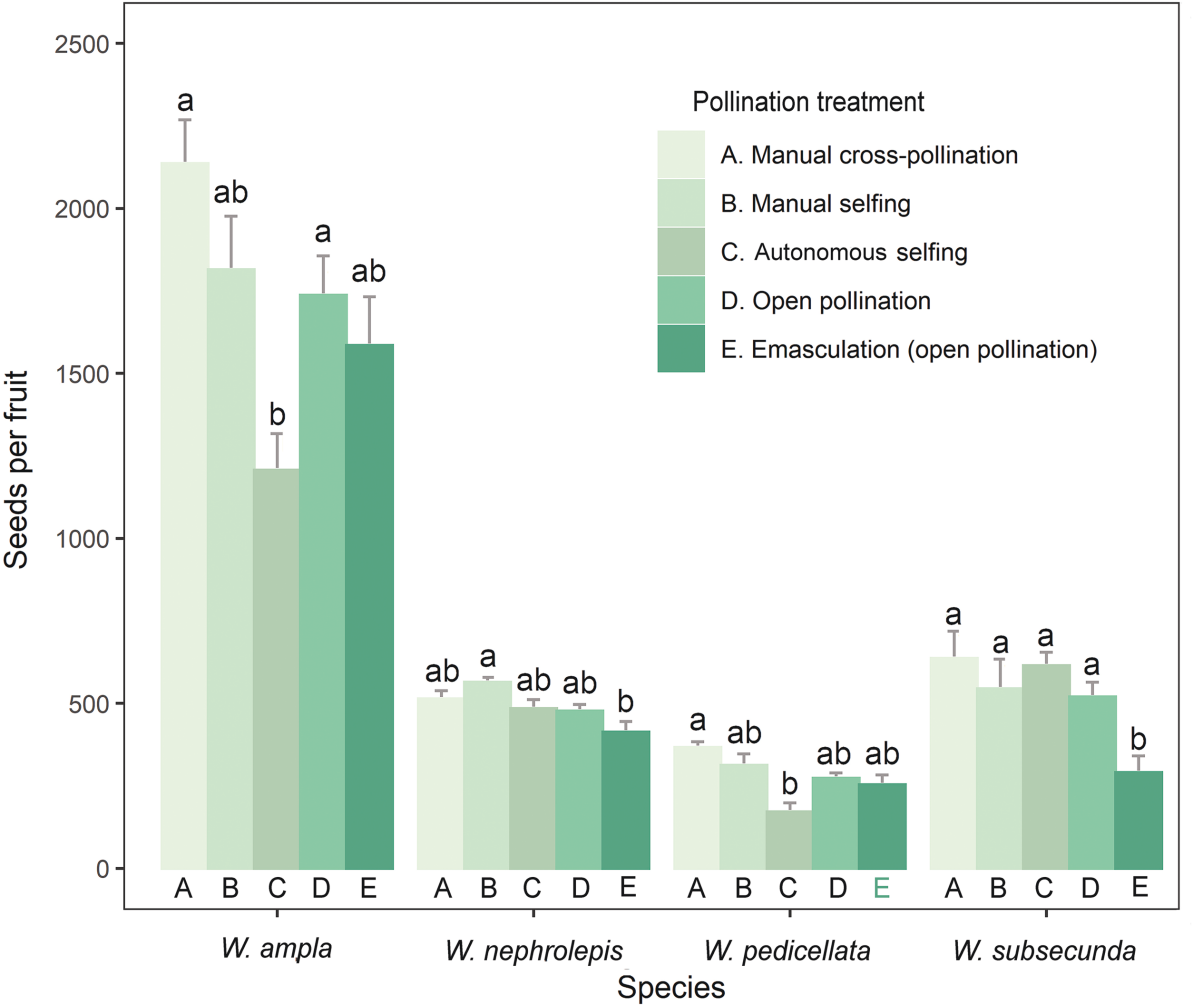
Seed set in four epiphytic *Werauhia* species (Bromeliaceae: Tillandsioideae) under different pollination treatments. Plants from a montane tropical forest, Cerros La Carpintera, Costa Rica. Bars are mean number of seeds per fruit and vertical lines are 1 SE. Different letters indicate significant differences between treatments per species after a Tukey test.

The average seed set per fruit did not significantly differ between manually self- and cross-pollinated fruits for each species (Fig. 2), supporting the high self-compatibility condition previously recorded using fruit-set data. Moreover, inbreeding depression effects were absent or low for seed production, with IDI values ranging from −0.10 to 0.15. Comparing the number of seeds produced by autonomous selfing versus controlled self-pollination revealed no statistically significant differences, indicating the high efficacy of selfing at the level of seed production. (Fig. 2). Similar amounts of seeds were developed in fruits from open and controlled cross-pollination (Fig. 2).

**Figure 3.**
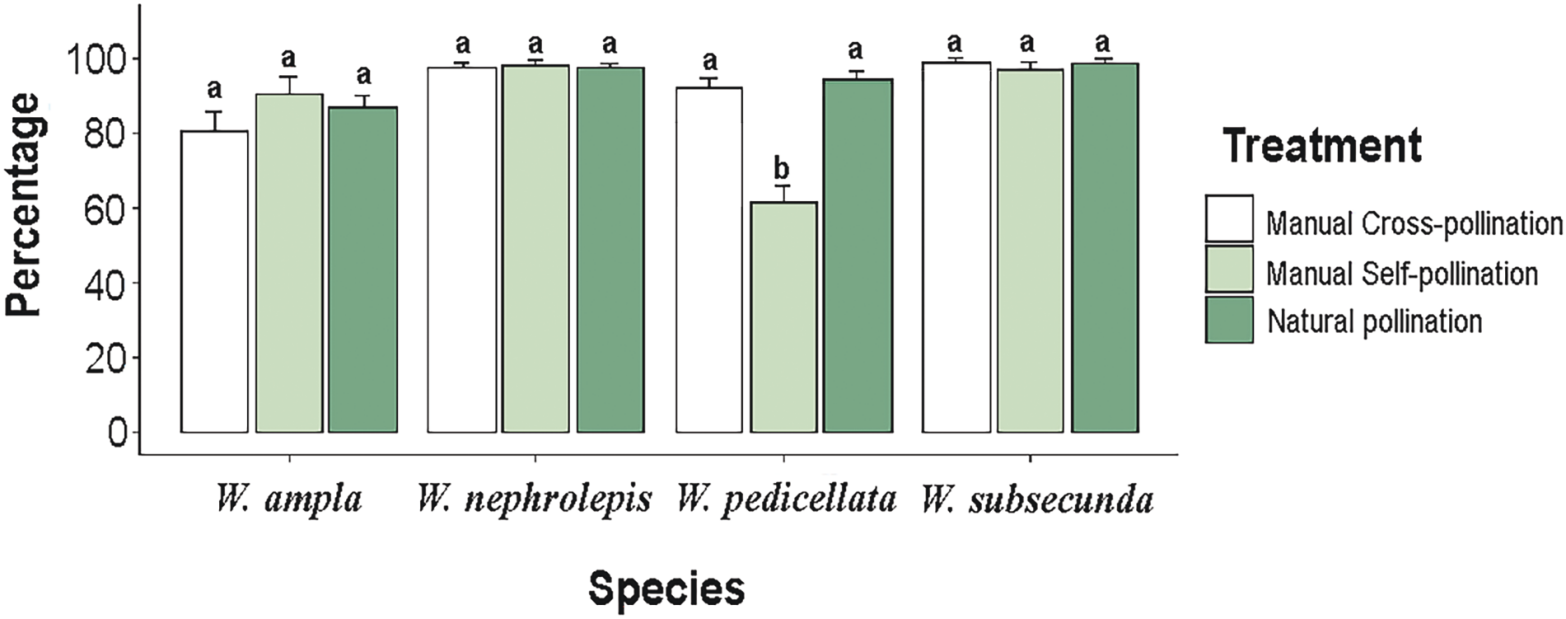
Seed germination capacity of progeny sired from hand self- and cross-pollination and open pollination in four species of *Werauhia* (Bromeliaceae: Tillandsioideae) from a montane forest, Cerros La Carpintera, Costa Rica. Data are mean germination percentages from 12 replicates of 40 seeds per treatment after two-months of monitoring. Vertical lines = 1 SE. Different letters indicate significant differences between treatments per species after a Wilcoxon’s test. The estimated values of the inbreeding depression index were for *W. ampla* = -0.13, *W. nephrolepis *= 0.00, *W. pedicellata* = 0.34 and *W. subsecunda* = 0.02.

Seed germination was high (>80%) and did not differ statistically between self-, cross-, and open pollinated seeds, except for *W. pedicellata*, which selfed seeds had a lower germination rate (Fig. 3). The studied species experienced null to low negative effects of inbreeding on their germination capacity, except for *W. pedicellata* (IDI value = 0.34). In all species, seedlings remained alive by the end of the experiment after two months of sowing.

### Reproductive assurance

**Figure 4.**
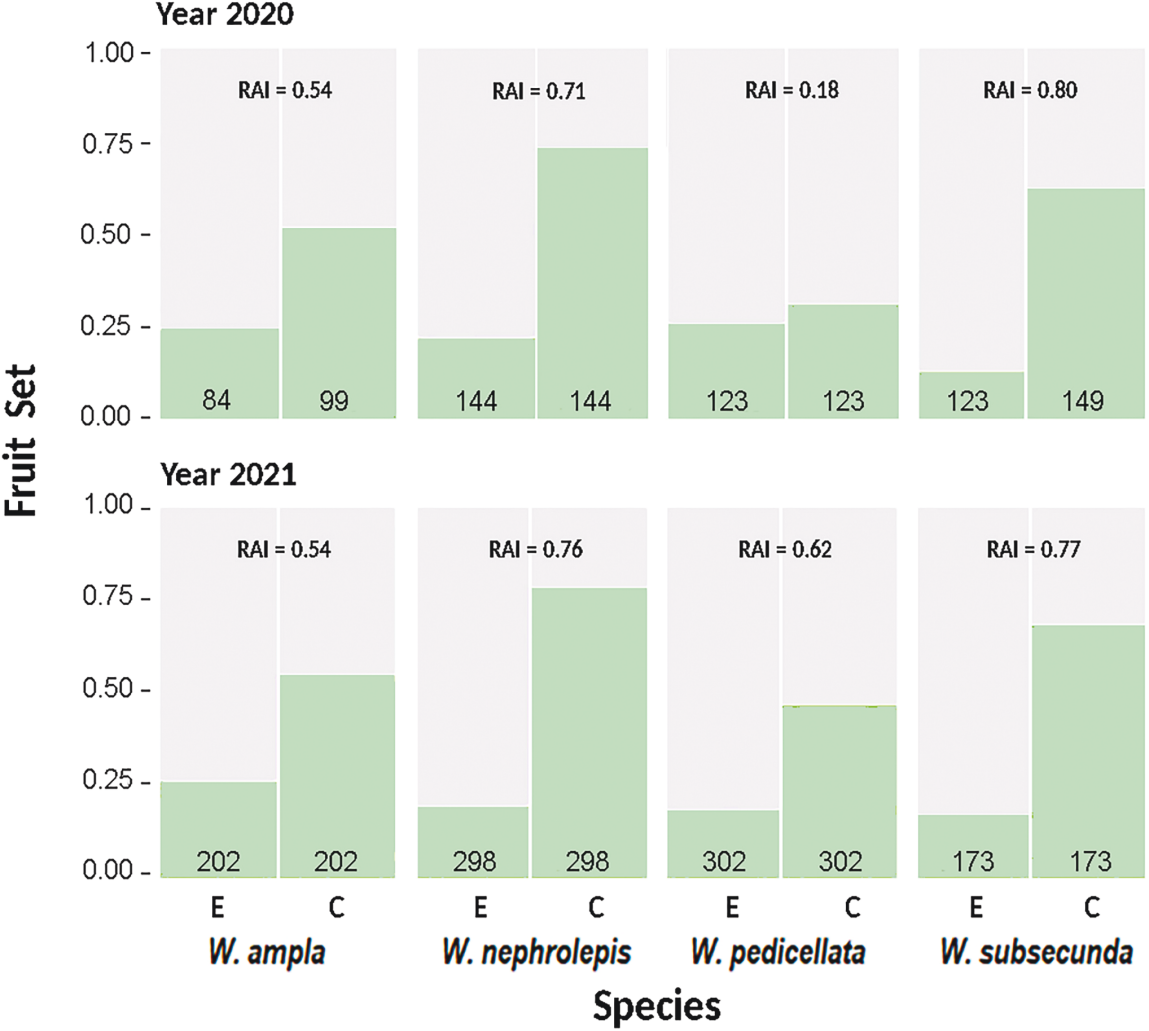
Fruit set from emasculated (E) and intact (C) flowers under open pollination conditions of four *Werauhia* (Bromeliaceae: Tillandsioideae) species in a montane forest, Cerros La Carpintera, Costa Rica. Bars represent the proportions of developed fruits per treatment in two consecutive reproductive seasons (2020 and 2021). The value of the *Reproductive Assurance Index* (RAI) is indicated for each species and year. The sample size (number of flowers) per treatment is indicated at the bottom of each column.

In all species and in both studied years, emasculated flowers developed fewer fruits compared to intact flowers (Figure 4). The GLM results indicated a significant effect of ‘treatment’, but neither ‘year’ nor their interaction did, except for *W. pedicellata* whose response was not consistent across years (Table 3, Figure 4). The odds ratios indicated that intact flowers capable of autonomous selfing had 3.4 times (in *W. ampla*) to nearly 12 times (in *W. subsecunda*) more chances of producing fruits than emasculated flowers that require pollinator visits (Table 3).

**Table 3.** Parameter estimates for the generalized lineal models on the production of fruits between emasculated and unmanipuled flowers under open-pollination conditions in four *Werauhia* species (Bromeliaceae: Tillandsioideae) in a montane forest, Cerros La Carpintera, Costa Rica. The reference categories are “emasculated” and “2020” for Treatment and Year, respectively.

Parameters by species	d.f.	Estimate	S.E.	Wald chi-square	*P*-value	Odds ratio	Confidence interval (95%)
*W. ampla*							
Intercept	1	−1.16	0.26	−4.54	<0.001		
Treatment	1	1.22	0.33	3.76	<0.001	3.40	1.80–6.44
Year	1	0.10	0.30	0.34	0.732	1.11	–
Treatment × year	1	0.03	0.39	0.09	0.929	1.03	–
Error	583						
*W. nephrolepis*							
Intercept	1	−1.29	0.20	−6.38	<0.001		
Treatment	1	2.32	0.28	8.37	<0.001	10.17	5.9–17.51
Year	1	−0.17	0.25	−0.68	0.498	0.84	–
Treatment × year	1	0.48	0.35	1.38	0.164	1.62	–
Error	880						
*W. pedicellata*							
Intercept	1	−1.09	0.21	−5.24	<0.001		
Treatment	1	0.28	0.29	0.99	0.321	1.33	–
Year	1	−0.44	0.26	−1.70	0.088	0.65	–
Treatment × year	1	1.12	0.34	3.28	0.001	3.07	1.57–6.01
Error	846						
*W. subsecunda*							
Intercept	1	−1.97	0.28	−7.16	<0.001		
Treatment	1	2.48	0.32	7.68	<0.001	11.96	6.34–22.53
Year	1	0.37	0.34	1.09	0.278	1.45	–
Treatment × year	1	−0.09	0.42	−0.21	0.831	0.92	–
Error	614						

The estimation of reproductive assurance indicated a contribution of autonomous selfing to fruit set from moderate to high (54–80%), except for *W. pedicellata* in 2020, whose fruit set was affected by herbivory (Figure 4). For seed production in 2021, the contribution of selfing to the number of seeds per capsule was low (RAI ≤ 0.14), except for *W. subsecunda* (RAI = 0.48) (Fig. 3).

## Discussion

The Bromeliaceae family exhibits a tendency towards selfing, but evidence of its potential adaptive value is lacking. In this study, we combined data from floral biology, pollination ecology, and breeding systems to demonstrate that selfing contributes significantly to the reproductive success of bromeliads. The studied *Werauhia* species from the Tillandsioideae subfamily showed a specialized pollination system that promotes out-crossing but experienced low visitation by nectar-feeding bats. The reduced events of cross-pollination were compensated by autonomous selfing that occurs at the end of the flower’s life and secures the plant´s reproductive success.

### Specialized pollination and mechanism of selfing

The studied *Werauhia* species conform to the traditional bat-pollination syndrome with nocturnal anthesis behaviour of flowers with dull coloration, emission of floral scents as chemical attractants and diluted nectar in high volume as reward (sensu Faegri and van der Pijl, 1979). The nocturnal video recording of bats visiting the flowers and the captured bats carrying pollen grains from the studied species confirmed this specialized pollination system.

Despite demonstrating unambiguous floral adaptations for cross-pollination, the breeding systems of the studied *Werauhia* were highly self-compatible and able to self-fertilize autonomously. The combination of incomplete protogyny and a lack of or variable herkogamy (in *W. ampla* and *W. pedicellata*) is likely what facilitates autonomous deposition of self-pollen on the stigma of the studied species. However, selfing did not appear to occur either before (prior) or during anthesis (competing) (*sensu*Lloyd and Schoen, 1992). When flowers were fully open, direct contact between stigma and anthers was prevented by the stigma’s distinctive cup-shaped lobes, which served to conceal the receptive area within (Brown and Gilmartin, 1989; Barfuss *et al.*, 2016). Also, the ventral torsion of the style near the stigma in *W. ampla* and *W. nephrolepis* may also reduce the chances of stigma-anther contact during anthesis. This feature of the style and stigma is found in other species of *Werauhia,* and the degree of torsion varies (Utley, 1983), but its potential significance to pollination has not been discussed previously.

Rather, we found that autonomous selfing in the studied Werauhias occurred at the end of the flower’s life. The pattern of flower senescence by which the corolla closes and forces the anthers with exposed pollen into contact with the still-receptive stigma corresponds to the mechanism of ‘corolla closure’ described by Goodwillie and Weber (2018). The stigmatic exudate that visibly accumulates in the stigma lobes of the studied species probably helps the pollen grains stick when the corolla closes. Following this evidence, self-pollination in the studied *Werauhia* would represent a mechanism of ‘delayed selfing’ (*sensu*Lloyd and Schoen, 1992), and it suggests that reproductive assurance rather than reproductive isolation is its primary benefit, as the latter would most likely select for earlier or preemptive selfing to prevent bats from depositing heterospecific pollen onto the stigmas (sensu Randle *et al.*, 2016).

Within the Bromeliaceae family, chiroterophily is present in subfamily Pitcairnioideae (*Pitcairnia*) but is better represented in Tillandsioideae, mainly in *Pseudoalcantarea* and *Vriesea,* and *Werauhia* is thought to be the genus with the greatest specialization in bat pollination (reviewed by Aguilar-Rodríguez *et al.*, 2019a). Fenster and Martén-Rodríguez (2007) suggested that specialized pollination is frequently associated with floral mechanisms to self-pollinate; however, several examples indicate that for bat pollination such association is weak. In a group of bat-pollinated gesneriads, Martén-Rodríguez and Fenster (2010) found they were unable to self-pollinate autonomously, while hummingbird-pollinated species exhibit high potential for autonomous selfing. Additional examples of neotropical chiropterophilous plants evidence the presence of self-incompatibility mechanisms or the inability to self-pollinate autonomously (e.g. Sazima and Sazima, 1978; Gibbs *et al.*, 1999; Gribel and Gibbs, 2002; Sazima *et al.*, 2003) suggesting that specialization in pollination and floral traits that promote selfing are not necessarily associated in an evolutionary context (Fenster and Martén-Rodríguez 2007). Thus, the high frequency of *Werauhia* species and bromeliads, in general, with specialized pollination systems and high selfing ability might be a particularity of this plant lineage.

### Pollinators and pollinator limitation

Two nectar-feeding bat species from the subfamily Glossophaginae (*Glossophaga soricina* and *Hylonycteris underwoodi*) represent the most probable pollinators of the studied species. Our census was limited in scope (two months), but according to a more extensive survey of the bat community (Durán, 2013), a third nectarivorous species (*G. commissarisi*) is present in our study site. Based on the frequency of captures, our data suggest that *Hylonycteris underwoodi* is likely the most important pollinator of the studied epiphytic bromeliads. This is a small nectarivorous bat distributed from Mexico to Panama in primary and older secondary forests and from sea level to 2640 m asl (Wilson and Mittermeier, 2019).

Plants with specialized pollination systems are prone to pollen limitation due to unpredictable visitation by their pollinators (Knight *et al.*, 2005; Martén-Rodríguez and Fenster, 2010). In chiropterophilous plants, bats are considered ‘good’ pollinators because they carry large amounts of pollen from different paternal genotypes and can disperse it over long distances (Fleming *et al.*, 2009). In spite of this, our evidence from camera traps suggests a low pollinator availability of nectar-feeding bats, with visitation ranging from none to 0.24 visits per night per plant. Data from other bat-pollinated bromeliads suggest varying but usually higher visitation rates; for instance, Aguilar-Rodríguez *et al.* (2019b) found no visitation to *Werauhia nutans* but up to 4.2 visits per flower per night in *Pseudalcantarea viridiflora*. While in *W. gladioliflora*, Tschapka and von Helversen (2007) observed 1–44 visits per flower per night. Bat visits to flowers can be quite fast (less than 0.5 s) and it is possible that camera traps have underestimated the visitation rate. However, our flower emasculation experiment, which resulted in low reproductive success (<26% fruit set), supports the idea of limited pollinator services in the studied epiphytic bromeliads.

The low pollinator visitation recorded may arise from the interaction of several ecological factors acting locally. A low diversity of pollinators has been associated with increased pollen limitation (Knight *et al.*, 2005). Species richness in nectar-feeding bat communities shows a decreasing pattern with respect to elevation (Fleming *et al.*, 2005), with fewer species in montane forests compared to lowland habitats. The absence of *Anoura geoffroyi* (Phyllostomidae) at the study site is notable since it is a nectarivorous species from montane forests and considered abundant throughout its distribution range (Ortega and Alarcón-D., 2008). In Costa Rica, however, it is an uncommon and rarely captured species, although it is apparently common in some localities (LaVal and Herrera-R., 2002; Wainwright, 2007). The lower diversity (three species) of pollinating bats in our research site, located at around 1700 m asl, compared to a Costa Rican lowland bat community with four nectarivorous species (Tschapka and von Helversen 2007), presumably plays a role in the limited visitation we recorded.

Low floral visitation may also be indicative of a low population density of pollinators. *Hylonycteris underwoodi* is a rare species that never occurs in dense populations and roosts in small groups of one to four individuals (Wilson and Mittermeier, 2019). In a lowland bat community, this bat species was unfrequently captured in mist nests and represented 4% of the captures (Tschapka and von Helverson, 2007). Similarly, in a previous bat inventory at our study site and with a sampling effort spanning a whole year (39 nights and 21 060 m^2^/h), Durán (2013) documented only five *H. underwoodi* individuals from a total of 142 captured bats (3.5% of the captures). Overall, the evidence strongly suggests that *H. underwoodi* has a low population density at our montane research site, which likely accounts for the observed low visitation frequency to bromeliad flowers. According to Fleming *et al.* (2005), nectarivorous bats density is probably low in most habitats; however, the aforementioned research by Tschapka and von Helversen (2007) also revealed a higher abundance of bats that frequently visited the flowers of *W. gladioliflora* in a lowland forest. This spatial variation in pollinator abundance may affect the efficiency of selfing as a reproductive assurance mechanism.

Factors related to habitat fragmentation may, in turn, affect the density of resident bat pollinators (Steffan-Dewenter and Tscharntke, 1999; Cunningham, 2000; Liu and Koptur, 2003; Knight *et al.*, 2005) and negatively impact pollination services. The studied montane forest is a medium-sized forest fragment (ca. 2.400 ha) loosely connected to major forested areas in the much larger Talamanca Mountain range. This condition may limit long-distance migration, affect the stability of the local population of *H. underwoodi,* or impede the establishment of other nectarivorous species such as *A. geoffroyi*. In addition, pollen grains of non-bromeliad plants recovered from bats suggest that inter-specific competition among co-flowering bat-pollinated plants may be a potential cause of decreased visitation. On the contrary, intra-specific competition for pollinators among sympatric *Werauhia* is likely low, since the investigated species exhibit a staggered flowering phenology in the study site (Cascante-Marín *et al.*, 2017). Furthermore, this phenological pattern may be an indicator that reproductive isolation is not the primary function of selfing but rather its reproductive assurance function.

### Reproductive assurance

The mechanism of autonomous delayed selfing of the studied *Werauhia* was key to their reproductive success, representing 54–80% of the total fruit set. Recording the time of selfing in bromeliad pollination studies is not a common practice (Cascante-Marín and Núñez-Hidalgo 2023), but the few studies that have reported delayed selfing in bat-pollinated and highly autofertile bromeliads belong to *Werauhia* species (Cascante-Marín *et al.*, 2005; Aguilar-Rodríguez *et al.*, 2019b). However, these studies did not assess its contribution to reproductive success.

The establishment and persistence of selfing are counteracted by the negative effects of inbreeding (Charlesworth and Charlesworth, 1987). Theoretically, the maintenance of selfing would occur when the adequacy of the selfed progeny surpasses that of outcrossed origin by a factor of W_S_/W_O_ > 0.5 (Herlihy and Eckert, 2002; Eckert *et al.*, 2006). We found that inbreeding depression at early stages of the progeny had low or null effects on the number of seeds (IDI-values ≤ 0.15) and germination capacity (IDI-values ≤ 0.34) of selfed seeds. This likely contributes to the maintenance of selfing in the studied *Werauhia* populations. However, life-time estimations of inbreeding depression would confirm or reject the positive effects of selfing and its evolutionary stability (Delmas *et al.,* 2014).

In floral emasculation experiments, reproductive assurance may be overestimated due to low visitation caused by modifications to flower attractiveness (Eckert *et al.,* 2006). In our case, anthers removal may have caused a minor alteration to the flower’s visual appearance, and we presume a non-significant effect since it has been demonstrated that nectar-feeding bats depend more on olfactory and acoustic cues when searching for nocturnal flowers (Gonzalez-Terrazas *et al.*, 2016). Also, bats appear to rely more on olfaction when flowers are situated against a complex background (Muchhala and Serrano, 2015), as is the case with epiphyte plants in the forest canopy.

Comparable data on manipulative experiments involving other bromeliads, as well as tropical plants in general, are severely lacking (see Eckert *et al.,* 2006; Busch and Delph 2012). Lasso and Ackerman (2004) found that emasculated flowers of the hummingbird-pollinated *Werauhia sintenisii* from the island of Puerto Rico experienced low pollinator visitation. The authors suggested the value of selfing in the reproduction of this species. Studies from temperate zone plants are more prevalent in the literature (e.g. Eckert *et al.,* 2006, Kalisz *et al.,* 2004; Moeller 2006; Brys and Jacquemyn 2011; Yang *et al.,* 2018; Teixido and Aizen 2019) and show that the effect of selfing on reproductive success exhibits temporal and spatial variation. The few studies on tropical plants have found that the contribution of selfing to reproductive assurance may vary among plants with different pollination systems in a group of gesneriads (Martén-Rodríguez and Fenster, 2010). It was also found that the contribution of selfing to the reproduction of the vine *Ipomoea hederacea* (Convolvulaceae) varied among reproductive seasons (Delgado-Dávila and Martén-Rodríguez 2021).

Selfing capacity and the degree of self-compatibility in Bromeliaceae are positively associated, with some of the variation explained by floral biology attributes such as anthers-stigma separation or herkogamy (Cascante-Marín and Núñez-Hidalgo, 2023). We found that *W. ampla* and *W. pedicellata* were highly self-compatible (SCI = 0.91 and 0.86, respectively) but exhibited lower selfing capacity (AFI = 0.53 and 0.54, respectively), which resulted in lower contribution to reproduction assurance. These differences in selfing capacity can be explained by the observed variation in herkogamy in the studied populations that may reduce the effectiveness of the selfing mechanism of corolla closure. Previous studies have shown that autofertility is correlated with variations in herkogamy; furthermore, this floral trait exhibits partitioning primarily between populations (Moeller, 2006). The variation of this floral trait is poorly documented in tropical plants, and it has been suggested that it can evolve rapidly in response to environmental changes affecting cross-pollination (Opedal *et al.,* 2017; Opedal 2018).

Ecological factors may offset the beneficial effects of selfing, as shown by the contrasting outcomes of the reproductive assurance estimation in *W. pedicellata*, despite its moderate selfing capacity (AFI = 0.54). This unexpected result can be explained by herbivory caused by larvae of a butterfly (Lepidoptera: Lycaenidae) that consumed early-developing capsular fruits in several plants during the 2020 season. Herbivory of reproductive structures may alter the reproductive success of plants, as documented in other bromeliad species (Cascante-Marín *et al.,* 2008; Orozco-Ibarrola *et al.*, 2015). This particular situation likely accounts for the lower fruit set in open pollination recorded in the first flowering season studied. This type of herbivory may result in complete loss of a plant´s inflorescence, as observed in plants kept in a greenhouse and field conditions.

## Concluding remarks

This study provides novel evidence of the function of delayed selfing as a reproductive assurance mechanism in the species-rich family Bromeliaceae, a plant lineage characterized by a tendency towards self-fertilization. The alternative hypothesis of selfing as a mechanism of reproductive isolation is not supported because of the non-overlapping flowering seasons previously reported for the investigated *Werauhia* species in the study site (Cascante-Marín *et al.*, 2017), which precludes heterospecific pollen transfer. Moreover, it has been demonstrated that delayed selfing is an ineffective barrier against hybrid fertilization (Brys *et al.*, 2016). A comprehensive study of potential isolation mechanisms will confirm this assumption.

We conclude that reproductive success in the studied *Werauhia* species is pollinator-limited due to the low visitation rate of its main bat pollinator. The delayed-selfing mechanism is strengthened by the lack of inbreeding depression and substantially contributes to reproductive success, compensating for the limited cross-pollination services provided by nectar-feeding bats. This selfing mode may be common among chiropterophilous bromeliads; however, the documented reproductive benefits may vary depending on the ecological context of pollination. Some reports of delayed selfing in predominantly ornithophilous bromeliad genera, such as *Tillandsia* (Orozco-Ibarrola *et al.*, 2015) and *Pitcairnia* (Wendt *et al.*, 2002), warrant further investigation to test whether selfing as a mechanism of reproductive assurance has also evolved in bromeliad lineages with other specialized pollination systems than chiropterophily.

The high prevalence of selfing in Bromeliaceae suggests a potential ecological and evolutionary advantage. Unveiling such benefits requires detailed studies combining floral biology, breeding systems, and pollination in bromeliads and other tropical plants. Manipulative experiments that encompass temporal and spatial variation in pollination conditions may help us understand the ecological factors that shape the effects of selfing in tropical plants.

## Supporting Information

The following additional information is available in the online version of this article –

Appendix. Raw data from experiments.

Table S1. Visitation data of nectarivorous bats to flowers of four epiphytic bromeliads from genus *Werauhia* (Bromeliaceae: Tillandsioideae) in a montane forest, Cerros La Carpintera, Costa Rica. Data from six video-camera traps from the flowering periods of 2019, 2020, and 2021.

Table S2. Number of pollen grains per plant species recovered from the six nectarivorous bats captured in a montane forest, Cerros La Carpintera, Costa Rica. Data from a sampling effort of 675 m^2^/h during eigth nights from January to February 2020.

Video S1. Time-lapse video of a flower senescence of *Werauhia ampla* (Bromeliaceae). Frame rate: 30 fps. Duration: 15 s.

Video S2. Time-lapse video of a flower senescence of *Werauhia subsecunda* (Bromeliaceae). Frame rate: 30 fps. Duration: 27 s.

Video S3. Time-lapse video of a flower senescence of *Werauhia nephrolepis* (Bromeliaceae). Frame rate: 30 fps. Duration: 16 s.

Video S4. Slow motion video (10×) of a bat visiting a nocturnal flower of *Werauhia ampla* (Bromeliaceae). Duration: 22 s.

Video S5. Slow motion video (10×) of a bat visiting a nocturnal flower of *Werauhia nephrolepis* (Bromeliaceae). Duration: 7 s.

Video S6. Slow motion video (10×) of a bat visiting a nocturnal flower of *Werauhia pedicellata* (Bromeliaceae). Duration: 10 s.

Video S7. Slow motion video (10×) of a bat visiting a nocturnal flower of *Werauhia subsecunda* (Bromeliaceae). Duration: 10 s.

plae011_suppl_Supplementary_Tables_S1-S2

plae011_suppl_Supplementary_Videos_S1

plae011_suppl_Supplementary_Videos_S2

plae011_suppl_Supplementary_Videos_S3

plae011_suppl_Supplementary_Videos_S4

plae011_suppl_Supplementary_Videos_S5

plae011_suppl_Supplementary_Videos_S6

plae011_suppl_Supplementary_Videos_S7

plae011_suppl_Supplementary_Appendix

## Data Availability

The data underlying this article are available in the article and in its online Supporting Information.
