# Supplementary material for: Selfing in epiphytic bromeliads compensates for the limited pollination services provided by nectarivorous bats in a neotropical montane forest
Source: AoB Plants. 2024 Feb 26;16(2):plae011. doi: 10.1093/aobpla/plae011 (PMC10944016; doi:10.1093/aobpla/plae011)
Supplement: Supplementary file 1 [file plae011_suppl_supplementary_tables_s1-s2.zip › plae011_suppl_Supplementary_Tables_S1-S2.odt]

Núñez-Hidalgo S. & Cascante-Marín A. 

Selfing in epiphytic bromeliads compensates for the limited pollination services provided by nectarivorous bats in a neotropical montane forest. AoB Plants (2024).

SUPPORTING INFORMATION

| TABLE  S1.  Visitation data of nectarivorous bats to plants of four epiphytic bromeliads from genus Werauhia (Bromeliaceae: Tillandsioideae) in a montane forest, Cerros La Carpintera, Costa Rica. Data from six video-camera traps from the flowering periods of 2019, 2020, and 2021. |
| --- |
| Species | Year | Plants monitored | Nights monitored | Flowers monitored | Recorded visits | Visits/plant /night |
| W. ampla | 2019 | 3 | 35 | 35 | 0 | 0.00 |
|  | 2020 | 8 | 72 | 72 | 0 | 0.00 |
|  | 2021 | 6 | 57 | 57 | 2 | 0.04 |
| W. nephrolepis | 2020 | 6 | 23 | 83 | 0 | 0.00 |
|  | 2021 | 11 | 67 | 300 | 16 | 0.24 |
| W. pedicellata | 2020 | 4 | 38 | 252 | 8 | 0.21 |
|  | 2021 | 6 | 67 | 554 | 5 | 0.07 |
| W. subsecunda | 2019 | 3 | 18 | 18 | 0 | 0.00 |
|  | 2020 | 3 | 26 | 26 | 2 | 0.08 |
|  | 2021 | 6 | 51 | 51 | 0 | 0.00 |
| Total |  | 56 | 454 | 1448 | 33 | 0.07 |

| TABLE S2. Number of pollen grains per plant species recovered from the six nectarivorous bats captured in a montane forest, Cerros La Carpintera, Costa Rica. Data from a sampling effort of 675 m2/h during eigth nights from January to February 2020. |
| --- |
| Bat species | Recovered from | Plant species |
| Werauhia ampla | Werauhia subsecunda | Ipomoea sp. | Merinthopodium  neuranthum | Calliandra calothyrsus | Schultesianthus leucanthus |
| Hylonicteris underwoodi | head | 508 | - |  | 21 | - | - |
| Glossophaga soricina | head | 7 | - | 60 | - | - | - |
| H. underwoodi | head | - | - | - | - | 3 | - |
|  | snout | - | - | - | - | 3 | - |
| H. underwoodi | head | 710 | - | 12 | 26 | - | - |
|  | snout | 156 | - | 3 | 70 | - | - |
| H. underwoodi | head | 5250 | - | - | 90 | - | 9853 |
|  | snout | - | 146 | - | 62 | - | 87 |
| H. underwoodi | head | - | - | 156 | - | - | - |
